# Climatic Conditions of the Month of June, 1854

**Published:** 1854-08

**Authors:** 


### EDITORIAL DEPARTMENT.

---

*Climatic Conditions of the month of June,* 1854.—During the month of June, 138 observations have been made in this city, at different hours of the day, upon the temperature, dew-point, and relative humidity of the atmosphere.

Of these, 108 have been made by ourself, at 302 Main street. The height of this station above the level of Lake Erie is sixty-five feet. Thirty observations were made at 2 o'clock, P. M.; 27 at 9, A. M.; 18 at 12, M.; 17 at 5, P. M., and 16 at 7, P. M.

The remaining 30 observations, as well as a daily record of the height of the barometer, were made by Mr. Wesley D. Allen, at the Merchants' Exchange, in the neighborhood of the docks, and at a height of twenty feet above the level of the Lake.

In connecting the results of these experiments with the epidemic constitution of the month, a few important facts become especially prominent The climate of Buffalo is, owing to its geographical situation, a very humid one. The relative humidity of the month has been universally high compared with other localities. But the dew-point, the height of which is the true test of the amount of aqueous vapor in the air, was during the early part of the month quite low. During the latter part it became higher, and has finally reached a point which indicates what Dr. Barton, of Louisiana, considers the principal cause of epidemics, combining great heat and humidity.

This cause is not thought to be a rapid one in its action, and only develops disease after a certain prolonged influence, and a lapse of time sufficient to allow a development by zymosis.

The month has been unusually healthy; only 160 deaths having occurred. Of these, eight were from cholera, and their relation to humidity will be indicated.

It will be noticed that the cases of cholera occurred in the latter half of the month, and that the majority of them happened within the last week. All but two of these were, perhaps, to be considered as imported cases. The fact, however, that two of them were indigenous, and that some five or six deaths occurred during the first two days of July, is sufficient evidence that a cholera predisposition was present. Only single cases occurred up to the 29th, on which day there were three deaths; and from that time up to the morning of the 3d, five additional deaths occurred; making eight deaths within four days.

Is there any evident ratio between the dew-point and the deaths? On the 22d the dew-point was at 65°, and its average for the remainder of the month was 63° 22. During the same period the relative humidity averaged 761.

Thus we have a period of more than a week characterized by high temperature, dew-point, and humidity, immediately preceding the development of the first important inroad of cholera. During this period a good number of cases recovered under treatment.

During the month, the prevalent wind was south-westerly, with only occasional and trivial variations. It was a subject of general remark that the air was chilly, even on our warmest days, that is; that a sensation of chilliness was immediately felt upon going into the shade.

There was but little rain. On the 6th, there was a heavy shower, lasting about an hour. At 8, P. M., on the evening of the 7th, there was a very heavy shower, lasting several hours. On the 8th, at noon, one lasting one hour. For a fortnight succeeding, no rain fell. On the 22d, commencing at 10, P. M., it rained four or five hours. On the night of the 28th, there was a very heavy rain, lasting six or seven hours.

We close with a general statement of the conditions of the various hours of the day.

BUFFALO, June, 1854.

9, *A. M.*—27 *Observations.*

| | |
|---|---|
| Average Temperature, | 69° 44 |
| " " of Wet Bulb, | 64° 88 |
| " Dew-point, | 58° 81 |
| " Humidity, | 726 |

12, *M.*—18 *Observations.*

| | |
|---|---|
| Average Temperature, | 70° 42 |
| " " of Wet Bulb, | 65 94 |
| " Dew-point, | 59 47 |
| " Humidity, | 723 |

2, *P. M.*—30 *Observations.*

| | |
|---|---|
| Average Temperature at Upper Station, | 71° 57 |
| " " " Lower " | 70 73 |
| " " of Wet Bulb at Upper Station, | 65 90 |
| " " " " " Lower " | 65 73 |
| " Dew-point at Upper Station, | 58 77 |
| " " " Lower " | 59 57 |
| " Humidity at Upper " | 684 |
| " " " Lower " | 728 |

5, *P. M.*—17 *Observations.*

| | |
|---|---|
| Average Temperature, | 73° 64 |
| " " of Wet Bulb, | 67 94 |
| " Dew-point, | 66 17 |
| " Humidity, | 671 |

7, *P. M.*—16 *Observations.*

| | |
|---|---|
| Average Temperature, | 73° 00 |
| " " of Wet Bulb, | 67 19 |
| " Dew-point, | 59 93 |
| " Humidity, | 697 |

*Average for the month at Upper Station.*

| | |
|---|---|
| Average Temperature, | 71° 57 |
| " " of Wet Bulb, | 66 31 |
| " Dew-point, | 60 63 |
| " Humidity, | 701 |

Whole number of Observations at Upper Station, 108. H.

